# Comparison between upper body and full underbody forced-air warming blanket in pediatric patients undergoing cardiovascular interventions under general anesthesia: a randomized controlled trial

**DOI:** 10.1186/s12871-025-03100-3

**Published:** 2025-05-21

**Authors:** Jung-Bin Park, Tae-Won Kim, Sang-Hwan Ji, Young-Eun Jang, Ji-Hyun Lee, Jin-Tae Kim, Hee-Soo Kim, Eun-Hee Kim

**Affiliations:** Department of Anesthesiology and Pain Medicine, Seoul National University Hospital, Seoul National University College of Medicine, 101 Daehak-ro, Jongno-gu, Seoul, 03070 Republic of Korea

**Keywords:** Hypothermia, Pediatric anesthesia, Cardiac intervention, Forced-air warming

## Abstract

**Background:**

Pediatric patients undergoing cardiovascular interventions outside the operating room are at high risk of perioperative hypothermia. We aimed to compare the effects of upper body and full underbody forced-air warming blankets on the time-weighted deviation of esophageal temperature outside the target range (36.5–37.5 °C) during general anesthesia.

**Methods:**

In this randomized controlled study, 88 children (age < 15 years) scheduled for elective cardiovascular interventions under general anesthesia were randomly assigned to the upper body (*n* = 44) or full underbody (*n* = 44) group. After the induction of anesthesia, warming blankets were applied and heated using a forced-air warmer to maintain an esophageal temperature of 36.5–37.5 °C. The primary outcome was the time-weighted average deviation of esophageal temperature outside the desired range, defined as the total deviation in temperature divided by the duration spent outside the target range. Secondary outcomes included use of additional warming or cooling methods, temperature trends, thermal comfort, and adverse events. Statistical comparisons were performed using t-tests or chi-square tests, with *p* < 0.05 considered significant.

**Results:**

The time-weighted averages of periods out of the desired temperature range were comparable between the two groups (upper body vs. full underbody, 0.213 ± 0.212 °C vs. 0.265 ± 0.277 °C; mean difference, 0.053; 95% confidence interval [CI], − 0.052 to 0.157; *p* = 0.318). The incidence of hyperthermia (> 37.5 °C) was 9.09% (upper body) and 0% (full underbody, *p* = 0.125). The duration of hypothermia (< 36.5 °C) was 58.82 ± 48.83 min (upper body) and 70.03 ± 53.20 min (full underbody; mean difference, 11.20 min; 95% CI, − 10.44 to 32.85; *p* = 0.318). The incidence rates of adverse events were 4.55% (upper body) and 15.91% (full underbody, *p* = 0.159).

**Conclusions:**

Both warming methods showed comparable time-weighted averages of temperatures outside the desired range, suggesting similar effectiveness. However, careful monitoring is essential to mitigate the risks of hyperthermia and skin-related complications and ensure patient safety during pediatric cardiovascular interventions.

**Trial registration number:**

NCT05349734 (registered at clinicaltrials, principal investigator: Hee-Soo Kim, registration date: April 26,2022).

**Supplementary Information:**

The online version contains supplementary material available at 10.1186/s12871-025-03100-3.

## Background

Patients undergoing cardiovascular interventions under sedation experience inadvertent hypothermia in 23.3% of cases [1]. Pediatric patients are at higher risk of hypothermia compared with adults because of their larger body surface area relative to body weight, relatively larger head, and immature thermoregulatory centers [2, 3]. Additionally, during cardiovascular interventions under general anesthesia, pediatric patients are typically exposed to the cold environment of a radiology intervention room for extended periods [2, 3]. Consequently, the deleterious effects of hypothermia, such as hypoventilation, relative anesthetic overdose, and increased oxygen demand, can be more pronounced in children undergoing cardiovascular interventions who have preexisting cardiopulmonary insufficiency with low peripheral perfusion and inadequate thermoregulation than their counterparts [2–5]. 

To prevent inadvertent perioperative hypothermia during general anesthesia, operating rooms are equipped with various devices, such as water-circulating mattresses, forced-air warming systems, and fluid warmers. However, in the intervention room, the presence of various equipment necessary for interventional procedures poses practical challenges in accommodating all thermoregulation devices typically used in operating rooms. Forced-air warming in a cardiac intervention room reduces hypothermia and improves the thermal comfort of patients under sedation [6]. However, the methods of warming blanket application considered effective significantly vary, depending on the type of surgery and patient’s position [7–10]. 

Recent studies have demonstrated the effectiveness of forced-air warming using underbody blankets [7, 9]. However, common issues with underbody blankets include incomplete inflation with warm air and wetting by antiseptic solutions, which are often undetected owing to surgical drapes [11]. Moreover, perioperative peripheral hypoperfusion during cardiac catheterization impedes heat dissipation and increases the risk of burn injuries [8, 12]. Considering these potential complications, upper body blankets may serve as a viable alternative. However, it remains unclear which blanket type—upper body or full underbody—is superior in maintaining intraoperative normothermia in pediatric patients undergoing cardiac catheterization.

In this study, we hypothesized that upper body blanket warming methods could offer better intraoperative normothermia compared with full underbody blanket warming methods in out-of-operating room settings for pediatric patients who underwent cardiac catheterization under general anesthesia. We aimed to investigate the effects of both warming methods on the time-weighted average of the period of out-of-desired temperature range.

## Methods

### Study design and ethics

This prospective randomized controlled study was conducted at Seoul National University Children’s Hospital between May 2022 and January 2024. This study was approved by the Institutional Review Board of Seoul National University Hospital (approval no. H-2005-016-1121, approval date: April 19, 2022, chairperson: Hyun-Hoon Jung) and registered at https://clinicaltrials.gov/study/NCT05349734 (registration number: NCT05349734, principal investigator: Hee-Soo Kim, registration date: April 26, 2022) prior to patient enrolment. This study adhered to the Declaration of Helsinki and guidelines outlined in the Consolidated Standards of Reporting Trials (CONSORT).

### Participants

Patients aged < 15 years scheduled for elective cardiac catheterization under general anesthesia at a single tertiary teaching hospital were enrolled in this study. The exclusion criteria included a scheduled procedure time of < 1 h, exposure of > 30% of the body surface area during cardiac catheterization, planned or attempted vascular access via the jugular vein, which requires exposure of the upper chest and shoulders, making it difficult to apply an upper body warming blanket during the procedure, thereby precluding study participation. Furthermore, exclusions were made for patients with contraindications to using an esophageal stethoscope, a corrected age of < 40 weeks, a pre-admission body temperature > 37.5 °C or < 35.5 °C, the presence of skin disease, a history of malignant hyperthermia, obesity (body mass index > 30 kg/m²), or any condition considered by the researchers to be unsuitable for study participation [13]. Detailed information about the study protocol was provided to the parents and patients, who provided written informed consent prior to catheterization.

### Randomization and blinding

The participants were randomly assigned to either the full underbody or upper body group. Randomization was conducted using an Internet-based response system (http://www.randomizer.org), with four blocks ensuring a 1:1 allocation ratio. Group assignments were concealed using sequentially numbered opaque sealed envelopes prepared by a clinical research nurse who was not involved in this study. The group assignment was revealed to the researcher by opening the envelope immediately after the participants entered the intervention room. This study was conducted in a double-blinded manner. While the anesthesiologists applying the warming device were unblinded, both participants and outcome assessors remained blinded throughout the study, including for the primary outcome and most postoperative evaluations.

### Intervention

For enrolled pediatric patients scheduled for cardiovascular interventions under general anesthesia, anesthesia was induced using standard methods. The children were kept dressed in the reception area, and the temperature of the intervention room was kept at 24 °C. Electrocardiography, pulse oximetry, noninvasive blood pressure or invasive blood pressure (if necessary) and patient state index (SEDLine^®^; Masimo Corp., Irvine, USA) were monitored. Propofol was used as the induction agent, and the dosage was determined at the discretion of the attending anesthesiologist. For maintenance of anesthesia, balanced anesthesia was administered using sevoflurane and a continuous infusion of remifentanil. Following endotracheal intubation or supraglottic airway insertion, an esophageal temperature probe was placed and continuously monitored, with recordings taken at 2-s intervals using the VitalRecorder [14]. The esophageal temperature probe was inserted according to the formula of Whitby and Dunkin, aiming to place its tip in the distal fourth of the esophagus, with its location confirmed via fluoroscopy during the procedure [15]. 

**Fig. 1:**
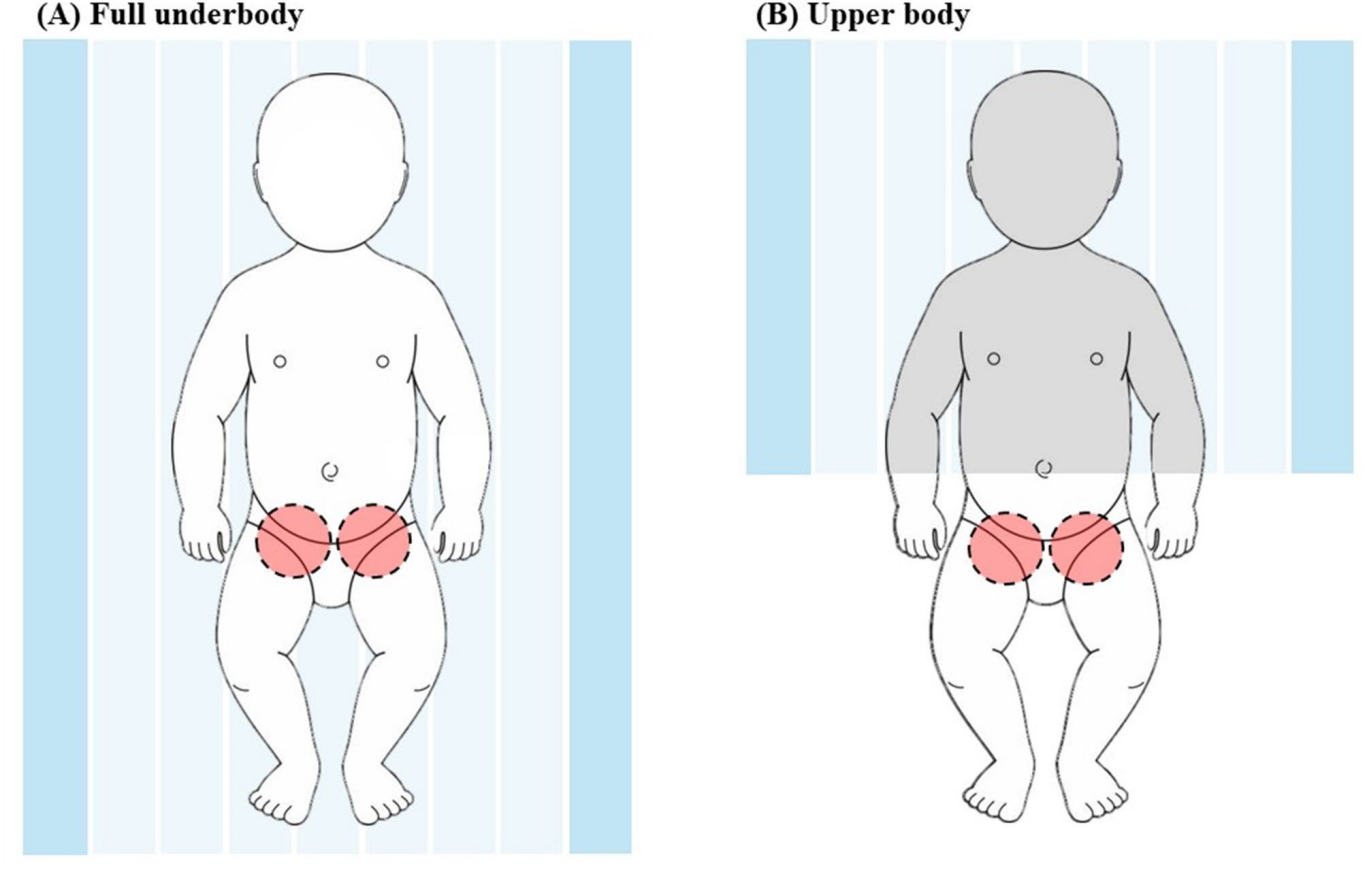
Application of warming blankets (**A**) Full underbody warming blanket positioned beneath the patient. (**B**) Upper body warming blankets covered from the navel to the upper limbs and head. The red circles indicate the locations of femoral vein puncture during cardiac catheterization

The full underbody blanket (3 M™ Bair Hugger™ Warming Blanket 55000 Pediatric Underbody; Eden Prairie, MN, USA) was positioned beneath the patient before the induction of anesthesia, whereas the upper body blanket (3 M™ Bair Hugger™ Warming Blanket 53700 Small Lower Body; Eden Prairie, MN, USA) was applied after the induction of anesthesia according to the group assignment (Fig. 1(A)). The upper body blanket, originally designed to cover the lower leg area, was modified to cover the navel to the upper limbs and head for the femoral vein puncture required for cardiac catheterization (Fig. 1(B)). A forced-air warmer (3 M™ Bair Hugger™ Model 775 Warming Unit) was not used during the induction of anesthesia and was initiated according to the study protocol after the insertion of the esophageal temperature probe, in order to ensure a consistent baseline core temperature measurement before warming. The proportion of the body surface area covered by the blanket was recorded using the Wallace rule of nines [16]. 

The desired temperature range during this study was specifically set to maintain esophageal temperature of 36.5–37.5 °C, which was referred to as normothermia for the purposes of this study [17, 18]. The temperature range for perioperative normothermia significantly varies according to the literature and expert opinions [2, 3, 19]. This range was selected because it aligns closely with our institution’s clinical practice targets. Mild hypothermia was defined when the measured core temperature was < 36.5 °C, and hypothermia was defined when the measured core temperature was < 36.0 °C for this study. Hyperthermia was defined as a core temperature > 37.5 °C. Warming was initiated if the core temperature was < 37.0 °C and ceased if the core temperature was > 37.0 °C.

**Fig. 2:**
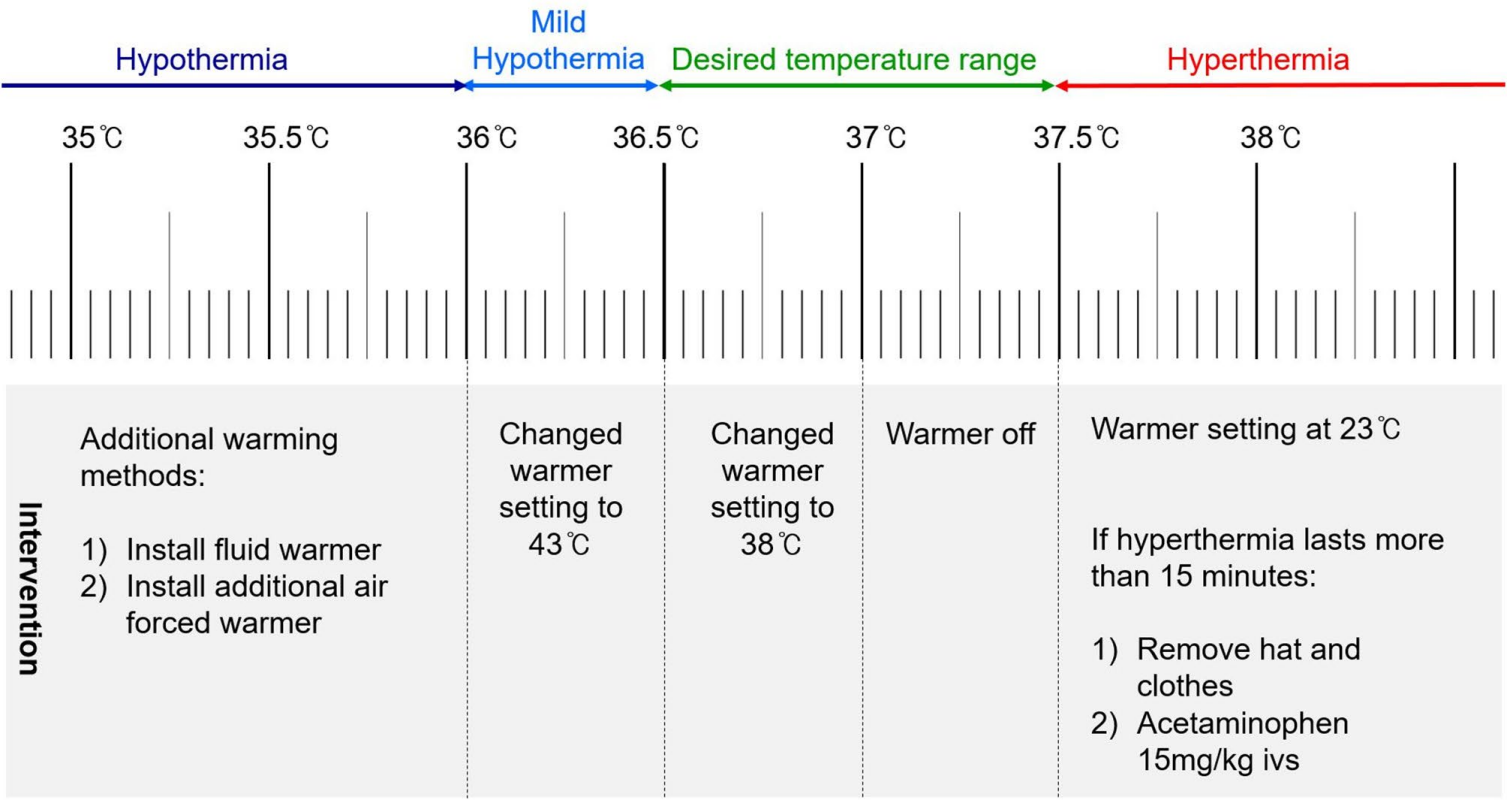
Study protocol

Our study followed an applied warming strategy as shown in Fig. 2. Forced-air warmer setting was adjusted to 38.0 °C when the patients’ core temperature was between 36.5 °C and 37.0 °C. The warmer was turned off when the core temperature was between 37.0 °C and 37.5 °C. If the core temperature exceeded 37.5 °C, the setting was lowered to 23 °C. In case of mild hypothermia, the warmer setting was increased to 43 °C. If hypothermia (< 36.0 °C) or hyperthermia (> 37.5 °C) persisted for > 15 min despite the use of a forced-air warmer, additional warming or cooling interventions were implemented at the discretion of the anesthesiologists. Any additional warming or cooling methods, such as the use of fluid warming devices or heated circuits during the procedure, were documented, including the duration of their use.

In the postanesthesia care unit, a blinded assessor evaluated the body temperature using an axillary thermometer and the shivering score using a Bedside Shivering Assessment Scale [20]. This scale categorizes shivering into four grades: no shivering noted on palpation of the masseter, neck, or chest wall (score 0); mild shivering localized to the neck or thorax (score 1); moderate shivering with gross movement of the upper extremities (score 2); and severe shivering with gross movements of the trunk and upper and lower extremities (score 3). For patients aged ≥ 6 years, postoperative thermal comfort was assessed by directly questioning the child regarding their perception of cold using a Numerical Rating Scale, where 0 indicated “not satisfied” and 10 “very satisfied.” Additionally, patient-reported shivering was evaluated by the responses to the question “Do you feel shivery?” posed to the child. During the hospital stay, adverse events and postoperative complications, including surgical site infections, pulmonary and gastrointestinal issues, morbidity, and mortality, were monitored.

### Outcomes

The primary outcome of this study was the time-weighted average of esophageal temperature values outside the desired range, defined as 36.5 °C to 37.5 °C, throughout the entire procedure duration. For each time point outside the normothermic range (36.5–37.5 °C), the absolute temperature deviation from the nearest boundary was calculated and summed across the procedure. This total deviation was then divided by the cumulative duration of out-of-range periods to yield the time-weighted average, expressed in degrees Celsius (°C) (Supplementary Fig. 1).

Secondary outcomes included changes in body temperature throughout the procedure, as well as the duration and time-weighted averages (absolute time and percentage) of periods categorized as mild hypothermia (< 36.5 °C), hypothermia (< 36.0 °C), and hyperthermia (> 37.5 °C). The proportion of body surface area covered by the warming blanket and that exposed to the surgical field were also recorded as descriptive variables to assist in interpreting the efficacy of the warming methods. The use and duration of any additional warming or cooling methods (e.g., fluid warmers, heated circuits) were recorded. The time required for extubation and the presence of shivering during extubation were assessed using the same BSAS score used in the postanesthesia care unit. Initial esophageal temperatures immediately after intubation and before extubation were collected. In the PACU, thermal comfort and patient satisfaction were assessed, along with the presence of postoperative shivering (BSAS) [20], and any adverse events related to warming devices or temperature probes. Postoperative complications monitored until discharge included respiratory events (desaturation, reintubation), cardiovascular instability (arrhythmia, hypotension), and skin-related adverse effects such as burns or redness.

### Sample size justification

Owing to the lack of existing studies on the use of full underbody and upper body blankets in pediatric patients undergoing cardiovascular interventions under general anesthesia, it is challenging to predict which method is more advantageous for maintaining perioperative temperature in children. A comparative study on underbody and overbody blankets in adults reported that after 2 h of surgery, the body temperatures were 37.1 °C and 36.8 °C, respectively, with a standard deviation (SD) of 0.5 [21, 22]. Using these data, a sample size of 44 patients per group was required to detect a significant difference between the two types of warm blankets, with an alpha error probability of 0.05 and a power of 0.8. Considering a 5% dropout rate, the target sample size was determined to be 92 participants.

#### Statistical methods

Normality was evaluated using the Shapiro–Wilk W-test. Data are presented either as means ± SDs or medians (interquartile ranges), depending on the characteristics of the variables. Categorical data were analyzed using Fisher’s exact and Pearson’s chi-squared test, whereas continuous data were assessed using the t-test and Mann–Whitney rank-sum test. A linear mixed-effects model (LMM) was applied to evaluate the differences in the time-dependent changes in esophageal temperature between the two study arms. The model included time as a continuous fixed effect, group (upper body vs. full underbody) as a categorical fixed effect, and subject as a random effect to account for repeated measures within the same patient. The temperature values at each time point were used as the dependent variable. To ensure a robust statistical evaluation, the analysis accounted for the correlation of repeated measurements within the same subject. The model was fit using Restricted Maximum Likelihood estimation. The time × group interaction term was also included to explore potential differences in the trajectory of temperature change between the groups. Statistical analysis were performed using IBM SPSS Statistics 22 (version 23.0; IBM Inc.). A two-sided *p*-value < 0.05 was considered statistically significant.

## Results

**Fig. 3:**
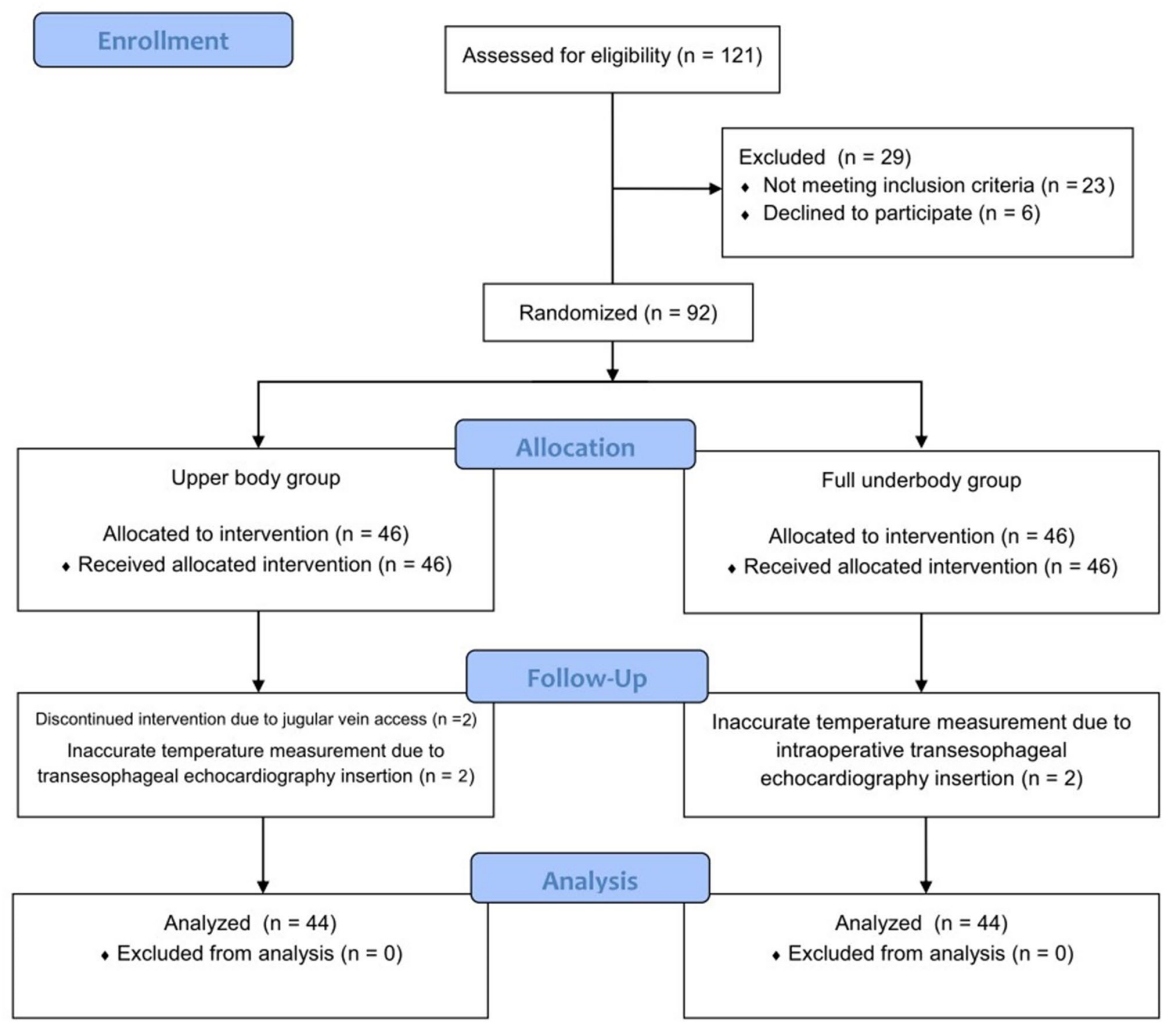
Consolidated standards of reporting trials (CONSORT) diagram

Ninety-two patients were included in this study, and four patients were excluded after enrollment. Follow-up was completed in 88 patients who were subsequently included in the final analysis (Fig. 3). One patient experienced an unexpected jugular vein puncture, which made the application of a warmer infeasible. Three patients required withdrawal of the esophageal stethoscope owing to the insertion of the transesophageal echocardiography device, resulting in their exclusion from the study. None of the patients exhibited hyperthermia (core temperature > 37.5 °C) or hypothermia (< 35.5 °C) at the beginning of anesthesia. The demographic data and types of cardiac interventions did not differ significantly between the two groups (Table 1). Additionally, intraoperative variables, such as administered fluids, transfusion, anesthesia duration, and estimated blood loss, were also comparable. During cardiac catheterization, the underbody group had a greater proportion of the body surface area covered compared with that of the upper body group (upper body vs. full underbody, 38.18 ± 13.55% vs. 49.43 ± 6.98%; mean difference, 11.26; 95% confidence interval [CI], 6.69 to 15.82; *p* < 0.001). The percentage of body surface area exposed to the surgical field was comparable.

**Table 1:** Baseline characteristics demographics and intraoperative data for the two study groups

	Upper body (*n* = 44)	Full underbody (*n* = 44)	*p*-value
Age (years)	6.8 ± 4.8	8.2 ± 5.2	0.198
Sex			0.201
F	19 (43.2%)	26 (59.1%)	
M	25 (56.8%)	18 (40.9%)	
Height (cm)	119.5 ± 34.1	127.2 ± 35.5	0.302
Body weight (kg)	28.7 ± 20.4	34.2 ± 22.7	0.237
Body surface area (m^2^)	1.0 ± 0.5	1.1 ± 0.5	0.240
Type of intervention			0.219
Device implantation (PDA, ASD, VSD)	12 (27.27%)	7 (15.91%)	
Balloon dilatation or valvuloplasty	18 (40.91%)	14 (31.82%)	
Radiofrequency catheter ablation	11 (25.00%)	20 (45.46%)	
Embolization of Collateral Vessels	3 (6.82%)	3 (6.82%)	
Operation time (min)	87.4 ± 52.5	99.5 ± 46.7	0.256
Anesthesia time (min)	115.9 ± 59.0	130.4 ± 53.2	0.231
Crystalloid (mL)	247.0 ± 367.3	320.7 ± 325.7	0.323
Cases requiring transfusion	1 (2.3%)	0	1
Estimated blood loss (mL)	11.8 ± 10.3	16.4 ± 15.6	0.11
The proportion of body surface area covered by the blanket (%)	38.18 ± 13.55	49.43 ± 6.98	< 0.001
The proportion of body surface exposure by surgical field (%)	5.47 ± 2.02	5.80 ± 2.25	0.472

ASD, atrial septal defect; PDA, Patent ductus arteriosus; VSD, ventricular septal defect

**Fig. 4:**
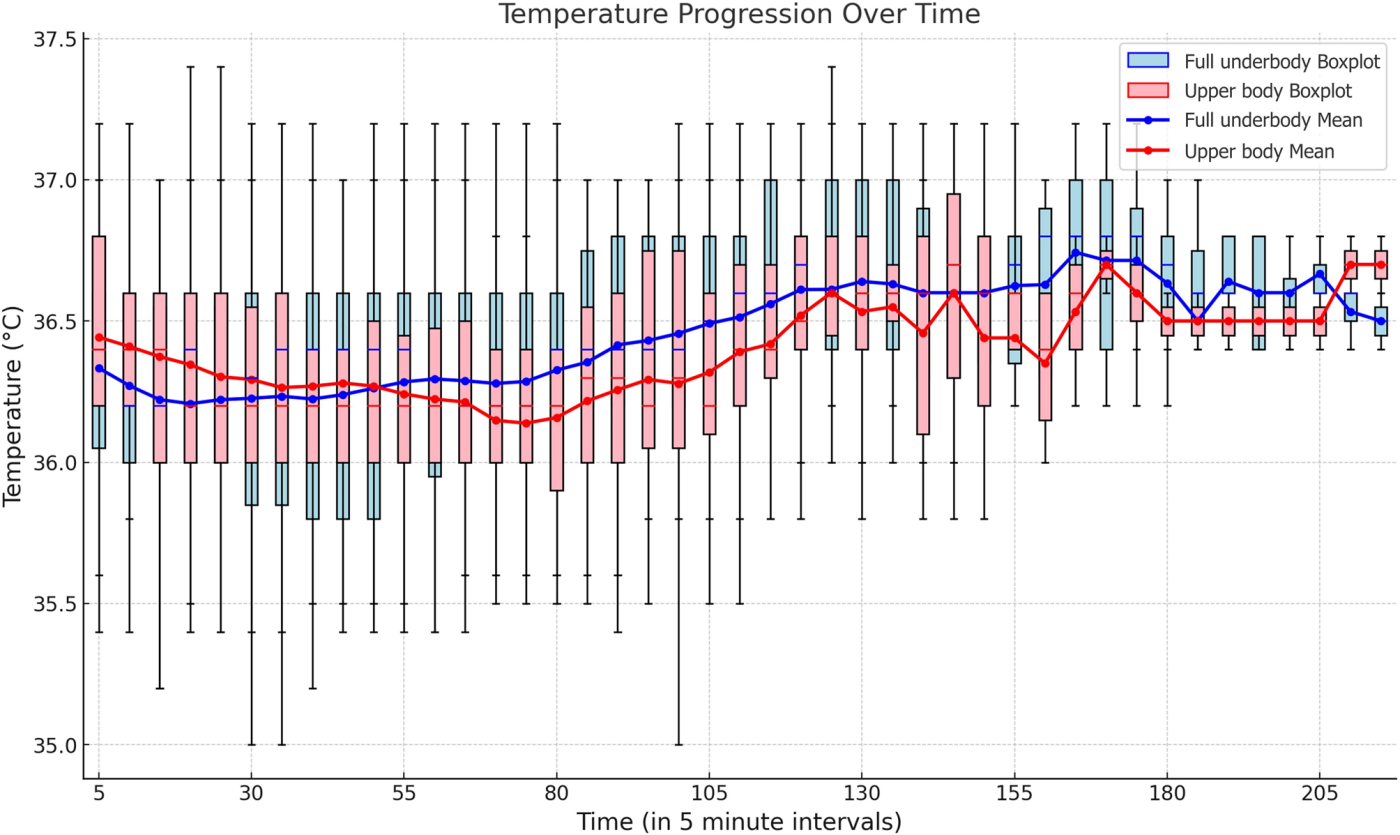
Comparison of core temperature progression in 5-min intervals in full underbody and upper body warming methods. The boxplots represent the distribution of temperature each 5-minute interval. Blue boxplots correspond to the full underbody warming method, while the red boxplots correspond to the upper body warming method. The median temperature is indicated by the colored lines within each box. The mean temperature progression over time is plotted as lines with markers. The blue line with circular markers represents the mean temperature for the full underbody warming method, while the red line with circular markers represents the mean temperature for the upper body warming method. There was no group difference observed at any of the time points

The primary outcome— the time-weighted average of temperature outside the desired range — was comparable between groups (0.213 ± 0.212 °C vs. 0.265 ± 0.277 °C; mean difference, 0.053; 95% CI, − 0.052 to 0.157; *p* = 0.318) (Table 2). The duration of these periods was also not different between the two groups (60.58 ± 47.43 min vs. 70.0 ± 53.20 min; mean difference, 9.44 min; 95% CI, − 11.92 to 30.81 min; *p* = 0.382). The number of cases with mild hypothermia (< 36.5 °C) and its duration were comparable between the two groups. Additional warming methods because of hypothermia (< 36.0 °C) were required in 14 (31.8%) patients in the upper body group and 10 (22.7%) patients in the full underbody group (odds ratio [OR], 0.63; *p* = 0.473). Hyperthermia (> 37.5 °C) was observed in four (9.1%) patients in the upper body group and in none of the patients in the full underbody group (*p* = 0.125). The time-weighted averages of the periods of hyperthermia were comparable (*p* = 0.185). A total of 13 (29.5%) patients in the upper body group and 9 (20.5%) patients in the full underbody group experienced an increase in body temperature > 37.0 °C, necessitating the cessation of warming (OR, 0.61; 95% CI, 0.23 to 1.63; *p* = 0.325). Warming was stopped for an average duration of 8.07 min in the upper body group and 5.32 min in the full underbody group (8.07 ± 15.21 min vs. 5.32 ± 14.33 min; mean difference, − 2.75 min; 95% CI, − 9.01 to 3.51 min; *p* = 0.385). The initial body temperature measured immediately after anesthesia induction, body temperature at emergence, and the difference between these two temperatures were not significantly different (*p* = 0.167, *p* = 0.745, and *p* = 0.122, respectively). Additionally, the highest and lowest body temperatures and the changes between these values did not differ between the two groups. Figure 4 shows the change in core temperature over time. A linear mixed-effects model showed that temperature increased significantly over time in both groups (*p* = 0.026). However, there was no statistically significant difference in the time interaction (*p* = 0.772), suggesting that the temperature change pattern over time did not significantly differ between the upper body and full underbody warming methods.

**Table 2:** Comparison of intraoperative thermal management and body temperature outcomes

	Upper body (*n* = 44)	Full underbody (*n* = 44)	Odds Ratio or Mean difference [95% CI]	*p*-value
Out of desired temperature range				
Number of cases	39 (88.64%)	42 (95.45%)	2.69 [0.49 to 14.69]	0.431
Duration (min)	60.58 ± 47.43	70.0 ± 53.20	9.44 [-11.92 to 30.81]	0.382
Time weighted average (°C)	0.213 ± 0.212	0.265 ± 0.277	0.053 [-0.052 to 0.157]	0.318
Mild hypothermia (< 36.5 °C)				
Number of cases	38 (86.36%)	42 (95.45%)	3.32 [0.63 to 17.43]	0.266
Duration (min)	58.82 ± 48.83	70.03 ± 53.20	11.20 [-10.44 to 32.85]	0.306
Time weighted average (°C)	0.207 ± 0.215	0.265 ± 0.277	0.059 [-0.047 to 0.164]	0.271
Hypothermia (< 36.0 °C)				
Number of cases	14 (31.81%)	10 (22.73%)	0.63 [0.24 to 1.63]	0.473
Duration (min)	13.52 ± 29.19	15.41 ± 31.36	1.89 [-10.95 to 14.73]	0.771
Time weighted average (°C)	0.180 ± 0.210	0.145 ± 0.185	0.035 [-0.086 to 0.156]	0.562
Hyperthermia (≥ 37.5 °C)				
Number of cases	4 (9.09%)	0 (0.00%)	Not applicable	0.125
Duration (min)	1.76 ± 7.24	0	-1.76 [-3.93 to 0.41]	0.111
Time weighted average (°C)	0.41 ± 1.92	0	-0.41 [-1.00 to 0.173]	0.160
Number of warming settings change				0.103
0	22 (50.00%)	27 (61.36%)		
1	18 (40.91%)	17 (38.63%)		
2	4 (9.09%)	0		
Intraoperative warming blanket stopped (≥ 37.0 °C)				
Number of cases	13 (29.55%)	9 (20.45%)	0.61 [0.23 to 1.63]	0.325
Duration (min)	8.07 ± 15.21	5.32 ± 14.33	-2.75 [-9.01 to 3.51]	0.385
Initially measured body temperature (℃)	36.46 ± 0.41	36.34 ± 0.42	-0.122 [-0.052 to 0.298]	0.167
Body temperature at emergence (℃)	36.47 ± 0.45	36.50 ± 0.39	0.030 [-0.210 to 0.151]	0.745
The change of body temperature throughout the procedure (℃)	0.007 ± 0.486	0.159 ± 0.426	0.152 [-0.346 to 0.042]	0.122
The highest body temperature (T_max_, ℃)	36.70 ± 0.41	36.71 ± 0.39	0.007 [-0.176 to 0.162]	0.936
The lowest body temperature (T_min_, ℃)	36.06 ± 0.44	36.05 ± 0.41	-0.007 [-0.173 to 0.186]	0.940
T_max_ - T_min_ (℃)	0.65 ± 0.41	0.66 ± 0.36	0.014 [-0.178 to 0.150]	0.869

**Fig. 5:**
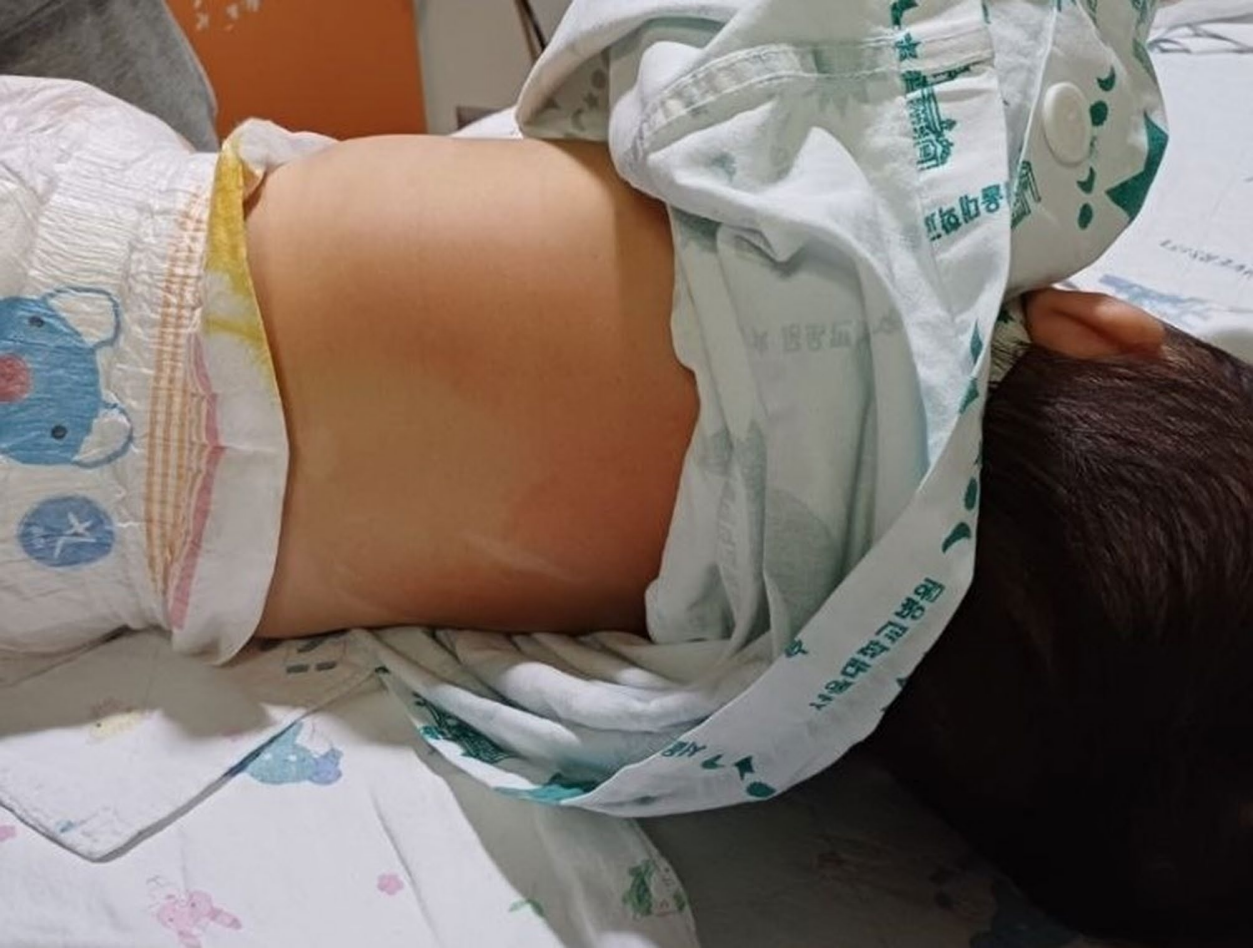
Example of skin-related adverse event observed after intraoperative warming. A photograph of a pediatric patient showing a linear erythematous mark with mild indentation on the back after use of a full underbody forced-air warming blanket. The mark resolved spontaneously without the need for treatment. This represents one of the skin-related adverse events recorded in the study

Patient-reported shivering, satisfaction scores, and extubation times were not significantly different between the two groups (Table 3). No shivering was observed during extubation in any patient. The number of patients who experienced any adverse events, including postoperative shivering, was comparable between the two groups (8 [18.18%] vs. 16 [36.36%]; OR, 2.57; 95% CI, 0.96 to 6.86; *p* = 0.094). Five (11.36%) patients in the full underbody group experienced skin-related adverse events, including complaints of redness (Fig. 5) and a stinging sensation in the legs, and one child had an indentation mark from the warmer. In the upper body group, one (2.27%) child had an indentation mark at the ear caused by the warmer, but there was no significant difference between the two groups (OR, 5.51; 95% CI, 0.62 to 49.28; *p* = 0.205). One child in the full underbody group experienced epistaxis because of the stethoscope. One patient in each group reported postoperative nausea and vomiting in the postanesthesia care unit.

**Table 3:** Postoperative outcomes

	Upper body (*n* = 44)	Full underbody (*n* = 44)	Odds ratio or Mean difference [95% CI]	*p*-value
Time to extubation	4.55 ± 3.38	4.20 ± 1.69	-0.34 [-1.47 to 0.79]	0.551
Postoperative shivering score at arrival in PACU^*^				0.730
3	1 (2.27%)	1 (2.27%)		
2	2 (4.55%)	4 (9.09%)		
1	4 (9.09%)	6 (13.64%)		
0	37 (84.09%)	33 (75.00%)		
Patient-reported shivering	6 (13.6%)	7 (15.9%)	1.20 [0.37 to 3.90]	0.929
Satisfaction score	5 [4, 8]	5 [4, 7]		0.306
Adverse events				
Total	8 (18.18%)	16 (36.36%)	2.57 [0.96 to 6.86]	0.093
Postoperative shivering	7 (15.91%)	11 (25.00%)	1.76 [0.61 to 5.07]	0.428
Skin-related	1 (2.27%)	5 (11.36%)	5.51 [0.62 to 49.28]	0.205
Stethoscope-related	0 (0.0%)	1 (2.27%)	Not applicable	1
Nausea/Vomiting	1 (2.27%)	1 (2.27%)	1 [0.06 to 16.51]	1

* Assessed using the Bedside Shivering Assessment Scale

PACU, post-anesthesia care unit

## Discussion

This study showed that upper body blanket warming methods could maintain body temperatures between 36.5 °C and 37.5 °C comparable with underbody blanket in children who underwent cardiac catheterization under general anesthesia in an out-of-operating-room setting when warmed by forced air. The occurrence of hyperthermia (> 37.5 °C) in four patients suggests the possibility of excessive warming with upper body blankets. Additionally, although the five skin-related adverse events in the full underbody group were not statistically significant, this finding may have potential clinical implications and should be carefully considered.

In this study, the upper body application demonstrated efficacy comparable with that of the full underbody application in maintaining body temperature, despite covering a smaller body surface area than the full underbody blanket. Although the effectiveness of warming blankets is generally attributed to the body surface area covered and efficiency of heat transfer, this may differ in pediatric patients during cardiac interventions. In such cases, the surgical site exposure is minimal (3.25–9%), procedures typically last approximately 1 h, and a relatively large body surface area can be covered with an upper body blanket (head, both arms, and chest) in children (24–44.5%) than in adults (22.5%) [16]. As a result, the upper body blanket covered a larger proportion of total body surface area than in adults; thus, the efficacy of upper body warming is more pronounced in these patients. Additionally, the upper body blanket offers the advantage of direct visual confirmation of proper air distribution, thereby ensuring effective warming.

Interestingly, hyperthermia only occurred in patients who received upper body warming. However, the overall incidence of hyperthermia in our study was lower than that reported in previous study, which have noted rates as high as 16.5%, particularly in younger children (< 2 years) and during prolonged procedures [8]. This discrepancy may be explained by several factors. Our procedures were performed in an out-of-operating-room setting, where the repeated use of cold contrast dye likely contributed to overall lower core temperatures. Additionally, our conservative and closely monitored warming strategy may have helped prevent excessive temperature elevation. In contrast, a previous study used a fixed warming setting of 40 °C for all patients and reduced the temperature only after core temperatures exceeded 37.3 °C, potentially contributing to a higher incidence of iatrogenic hyperthermia [8]. 

Furthermore, thermoregulation during anesthesia in children differs from that in adults. Thermoregulatory responses may lead to a significant increase in core temperature even under stable ambient conditions, due to peripheral and central vasoconstriction and increased metabolic heat generation within a smaller central compartment volume [23]. In our study, the upper body blanket group showed slightly more frequent adjustments to the warming device, though not statistically significant. This may suggest a latency in response to rising temperature, contributing to transient iatrogenic hyperthermia. Moreover, the upper body blanket covers a larger proportion of the child’s core and head, which may lead to more effective heat retention. These findings emphasize the need for continuous temperature monitoring and timely warming adjustments when using upper body warming devices in children.

Although we did not directly measure power consumption, it is likely that the upper body blanket—being smaller in surface area—requires less energy to operate compared to the full underbody blanket. If the two devices demonstrate comparable efficacy in temperature maintenance and similar rates of adverse events, then lower energy consumption could represent a meaningful advantage in terms of resource efficiency. This potential benefit warrants further investigation in future studies that assess not only clinical outcomes but also environmental and cost-related implications of warming device selection.

In this study, we observed a relatively higher number of skin-related adverse events, particularly in the full underbody blanket group. These included subjective complaints such as redness, stinging sensations, and minor indentation marks. While these events were not clinically severe, their frequency warrants attention. Several prior studies have reported skin complications associated with forced-air warming, though the reported incidence varies widely [24–28]. One possible explanation for the higher frequency in our study is our active surveillance approach: even mild or self-reported symptoms were carefully documented. Additionally, underbody blankets are typically covered by surgical drapes, which can obscure visual detection of incomplete inflation, excessive heating, or early skin changes. Pediatric patients undergoing cardiac catheterization are particularly vulnerable due to reduced peripheral perfusion from femoral artery cannulation, low cardiac output, and compensatory vasoconstriction [12]. The combination of localized hypoperfusion and obstructed heat dissipation may increase the risk of thermal injury [24]. These findings suggest that cutaneous complications from forced-air warming may be more common than previously assumed, especially in pediatric populations, and highlight the need for standardized monitoring and reporting practices.

This study has some limitations. First, it was conducted at a single tertiary center, which may have limited the generalizability of the results. Second, the sample size was calculated using adult data due to limited pediatric references. This may have reduced the power to detect subtle differences between groups. Future studies should use pediatric-specific effect sizes and larger samples to ensure adequate power. Third, the normothermic range used in this study (36.5–37.5 °C) was defined according to institutional protocols derived from neonatal guidelines. However, this range may be narrower than those recommended in other perioperative guidelines, such as those from NICE or the German Society of Anesthesiology, which often define normothermia as ≥ 36.0 °C [29, 30]. Consequently, temperatures slightly below 36.5 °C in school-aged children may not necessarily indicate clinically meaningful hypothermia. Similarly, our definition of hyperthermia as > 37.5 °C, while consistent with institutional practice, may have been overly strict and led to the classification of minor, physiologically normal temperature elevations as hyperthermia—particularly in children with intact thermoregulatory responses. Fourth, shivering was assessed using the Bedside Shivering Assessment Scale (BSAS), which has not been validated for use in pediatric or perioperative settings. Although it was used for practicality, we did not account for potential non-thermal causes of shivering, such as opioid-related effects. These limitations highlight the need for validated, pediatric-specific tools and further investigation into multifactorial causes of postoperative shivering. Finally, postoperative temperature in the PACU was measured via axillary thermometry, as maintaining the esophageal probe postoperatively was not feasible. Although axillary temperature is less accurate and not recommended as a substitute for core temperature, it was selected based on clinical feasibility [13]. These limitations may have introduced some degree of measurement bias; however, they did not affect the primary intraoperative outcome, which was based on continuously monitored esophageal temperature. In addition, prewarming was not performed prior to anesthesia induction, which may have contributed to early redistribution hypothermia.

## Conclusions

In conclusion, both the upper body and full underbody warming methods are effective in maintaining intraoperative normothermia in pediatric patients undergoing cardiac catheterization under general anesthesia. Continuous monitoring and proper use of warming devices are essential to minimize the risk of skin-related adverse events and inadvertent hyperthermia. Future studies are required to optimize warming methods in cardiac intervention rooms, explore the potential benefits of different warming strategies on long-term outcomes, and improve perioperative care for pediatric patients.

## Electronic supplementary material

Below is the link to the electronic supplementary material.

Supplementary Material 1

Supplementary Material 2

## Data Availability

The datasets used and/or created during this investigation can be obtained from the corresponding author with a reasonable request.

## Abbreviations

ASD: Atrial septal defect
CI: Confidence interval
CONSORT: Consolidated standards of reporting trials
OR: Odds ratio
PACU: Post-anesthesia care unit
PDA: Patent ductus arteriosus
SD: Standard deviation
VSD: Ventricular septal defect
