# Supplementary material for: Comparison between upper body and full underbody forced-air warming blanket in pediatric patients undergoing cardiovascular interventions under general anesthesia: a randomized controlled trial
Source: BMC Anesthesiol. 2025 May 21;25:254. doi: 10.1186/s12871-025-03100-3 (PMC12093743; doi:10.1186/s12871-025-03100-3)

**Supplementary Figure**. Graphical illustration of the primary outcome: the calculation method for the time-weighted average of esophageal temperature outside the desired range (36.5°C to 37.5°C). Temperature measurements were recorded every 2 seconds. For each data point outside the target range, the deviation (Δt) was multiplied by the measurement interval (2 seconds). The sum of these products (i.e., the total area outside the range) was then divided by the total time spent outside the normothermic range, expressed in degrees Celsius (°C).


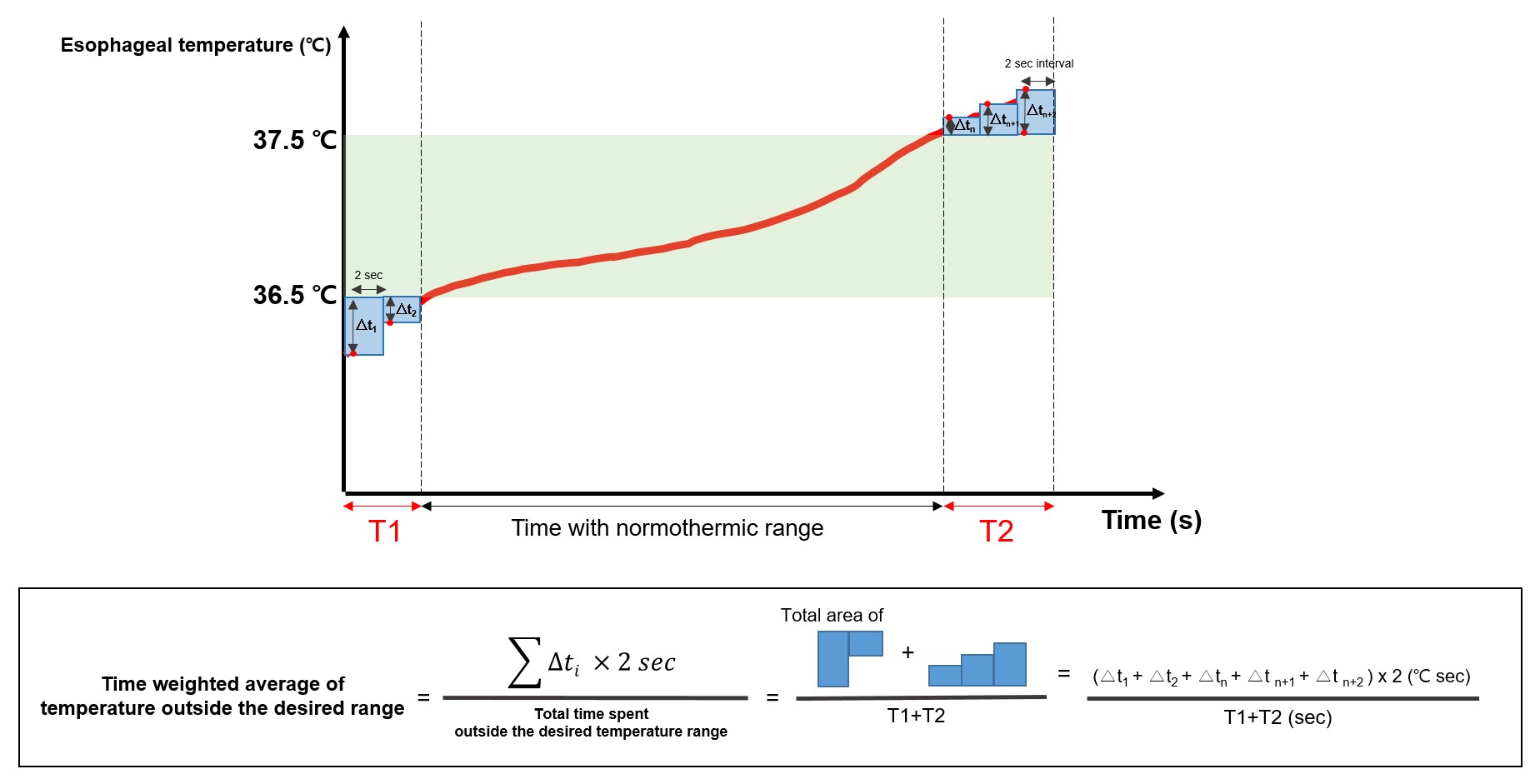

Supplement: Supplementary file 2 — Supplementary Material 2 [file 12871_2025_3100_MOESM2_ESM.docx]
